# Ferredoxin 2 Is Critical for Tumor Suppression and Lipid Homeostasis but Dispensable for Embryonic Development

**DOI:** 10.1016/j.ajpath.2024.12.002

**Published:** 2024-12-26

**Authors:** Jin Zhang, Yanhong Zhang, Shakur Mohibi, Vivian Perng, Miranda Bustamante, Yang Shi, Kenichi Nakajima, Mingyi Chen, Xinbin Chen

**Affiliations:** ∗ Comparative Oncology Laboratory, Schools of Veterinary Medicine and Medicine, University of California at Davis, Davis, California; † Department of Pathology, University of Texas Southwestern Medical Center, Dallas, Texas

## Abstract

Ferredoxin 1 and 2 (FDX1/2) constitute an evolutionarily conserved FDX family of iron-sulfur cluster–containing proteins. FDX1/2 are cognate substrates of ferredoxin reductase and serve as conduits for electron transfer from NADPH to a set of proteins involved in biogenesis of corticosteroids, hemes, iron-sulfur cluster, and lipoylated proteins. Fdx1 is essential for embryonic development and lipid homeostasis. Herein, *Fdx2*-deficient mice were generated to explore the physiological role of FDX2. Interestingly, unlike *Fdx1*-null embryos, which were dead at embryonic day 10.5 to 13.5, *Fdx2*-null mice were viable. Both *Fdx2*-null and *Fdx2*-heterozygous mice had a short lifespan and were susceptible to spontaneous tumors and steatohepatitis. Moreover, FDX2 deficiency increased, whereas overexpression of FDX2 decreased cytoplasmic accumulation of lipid droplets. Consistently, FDX2 deficiency led to accumulation of cholesterol and triglycerides. Mechanistically, FDX2 deficiency suppressed expression of cholesterol transporter ATP-binding cassette transporter A1 (ABCA1) and activated master lipid transcription regulators sterol regulatory element-binding proteins 1/2, thus leading to altered lipid metabolism. Untargeted lipidomic analysis showed that FDX2 deficiency led to altered biosynthesis of various lipid classes, including cardiolipins, cholesterol, ceramides, triglycerides, and fatty acids. In summary, these findings underline an indispensable role of FDX2 in tumor suppression and lipid homeostasis at both cellular and organismal levels without being a prerequisite for embryonic development.

Ferredoxins (FDXs) are ubiquitous, small proteins that play a critical role in a plethora of fundamental biological processes across all domains of life.^1^ These proteins are characterized by the presence of iron-sulfur clusters (ISCs), which play a pivotal role in facilitating electron transfer from NADPH, mediated by ferredoxin reductase (FDXR), to various biological systems. These systems encompass diverse biological processes, including ISC synthesis, corticosteroidogenesis, bile acid production, and vitamin metabolism.

Within mammalian cells, two distinct members of the ferredoxin family, FDX1 and FDX2, are prominently localized to mitochondria.^2^ Despite sharing substantial homology in both primary sequence and three-dimensional structure, FDX1 and FDX2 exhibit pronounced differences in substrate specificity and function.^3^ FDX1 primarily donates electrons to several cytochrome P450 systems within mitochondria. Its involvement spans a wide array of vital processes, including corticosteroid hormone synthesis from cholesterol, bile acid production, the addition of heme α to cytochrome *c*, participation in vitamin A/D metabolism, and lipoylation of tricarboxylic acid cycle enzymes.^3^^,^^4^

Since its discovery a decade ago, FDX2 has emerged as a pivotal player in mitochondrial biology, taking center stage in the formation of ISCs that are essential for various cellular processes.^3^ By serving as an electron donor to the iron-sulfur cluster assembly complex at two critical steps, FDX2 actively participates in the intricate process of 2Fe-2S as well as 4Fe-4S cluster biogenesis, thereby influencing critical cellular functions. Notably, as several critical metabolic and lipid synthesis enzymes contain an ISC, it points to a vital role of FDX2 in cellular lipid metabolism, a concept that has not been explored before.

Lipids represent a diverse class of biomolecules essential for a wide range of cellular processes, including membrane structure, energy storage, and intracellular signaling pathways.^5^ Key lipid species, such as triacylglycerides (TAGs), cholesterol, cardiolipins, and phospholipids, play pivotal roles in maintaining normal cellular physiology. Disruptions in lipid homeostasis are implicated in various disease pathologies, including cancer, neurodegenerative disorders, and cardiovascular diseases.^5^, ^6^, ^7^ Altered lipid metabolism is also closely associated with systemic inflammation and conditions like nonalcoholic steatohepatitis.^7^, ^8^, ^9^, ^10^

The sterol regulatory element-binding proteins (SREBPs), namely SREBP1/2, are recognized as master regulators of lipid metabolism and frequently implicated in perturbations of lipid homeostasis.^11^^,^^12^ Under normal physiological conditions, when intracellular cholesterol levels dip, SREBP1/2 undergo proteolytic cleavage, releasing their N-terminal domains. These domains subsequently translocate to the nucleus, where they activate a battery of genes, such as stearoyl-CoA desaturase 1 (SCD1), fatty acid synthase (FASN), and 3-hydroxy-3-methylglutaryl coenzyme A (HMG-CoA) reductase, responsible for lipid metabolism.^11^, ^12^, ^13^ Notably, in pathologic states, various mechanisms lead to the constitutive activation of SREBP1/2, resulting in aberrant lipid production and accumulation.^11^^,^^12^

SREBP-1 exists in two isoforms, SREBP-1a and SREBP-1c, both transcribed from the SREBF1 gene via separate promoters. SREBP-2, on the other hand, is encoded by the SREBF2 gene. Functionally, SREBP-1c predominantly governs the genes involved in fatty acid and TAG synthesis, whereas SREBP-2 primarily regulates genes associated with the cholesterol synthetic pathway, including the mevalonate pathway.^14^ SREBP-1a displays overlapping functions with both SREBP-1c and SREBP-2.

FDXR and FDX1 play an essential role in mammalian embryonic development and lipid metabolism.^15^, ^16^, ^17^ As FDX2 is a substrate of FDXR involved in the formation of ISCs, which are essential cofactors of critical metabolic and lipid synthesis enzymes, we reasoned that FDX2 might be involved in regulation of lipid metabolism in cells as well as in mice and thus regulate mouse development. However, unlike *Fdxr*^*–/–*^ and *Fdx1*^*−/−*^ mice, *Fdx2*^*−/−*^ mice were viable. On the other hand, both *Fdx2*^*+/–*^ and *Fdx2*^*−/−*^ mice had a short lifespan and were prone to spontaneous tumors and steatohepatitis. Importantly, FDX2 was involved in regulation of lipid metabolism via the ABCA1-SREBP1/2 pathways. Finally, loss of FDX2 led to aberrations in multiple lipid classes.

## Materials and Methods

### *Fdx2* Mutant Mouse Model

The use of animals and the study protocols used in this article were approved by the University of California at Davis Institutional Animal Care and Use Committee. *Fdx2*^*+/−*^ mice were generated by the Japan RIKEN BioResource Research Center (Tsukuba, Japan). The primers used to genotype the *Fdx2*–wild-type (WT) and Fdx2-knockout (KO) alleles were 5′-GAAGTCCAGCTAGATCCCAGTGCAT-3′ (forward), and 5′-CTCACACGTCCCGGGGCAGCTCCGG-3′ (reverse).

### Mouse Embryonic Fibroblast (MEF) Isolation

MEFs were isolated from 12.5 to 13.5 days post-coitum mouse embryos, as described previously.^18^ To generate WT, *Fdx2*^*+/−*^*,* and *Fdx2*^*−/−*^ MEFs, *Fdx2*^*+/−*^ mice were interbred. The MEFs were cultured in Dulbecco's modified Eagle's medium supplemented with 10% fetal bovine serum (Hyclone Laboratories, Logan, UT), 55 μmol/L β-mercaptoethanol, and 1× nonessential amino acids solution (Cellgro, Manassas, VA).

### Cell Culture

SCp2 mouse mammary epithelial cells were a generous gift from Pierre-Yves Desprez (California Pacific Medical Center Research Institute, San Francisco, CA) and were maintained as previously described.^19^ The mouse normal liver FL83B cells and the human cancer cell lines (HCT116, MIA-PaCa2, and HLF) were obtained from ATCC (Manassas, VA) and used below passage 20. Huh7, HLF, FL83B, HCT116, and MIA-PaCa2 cells were cultured in Dulbecco's modified Eagle's medium (Invitrogen, Waltham, MA) supplemented with 10% fetal bovine serum (Hyclone Laboratories).

#### siRNA Transfection

Scrambled siRNA and siRNAs against FDX2 (siFDX2#1 and siFDX2#2) were purchased from Dharmacon (Lafayette, CO). For siRNA transfection, Lipofectamine RNAiMAX Reagent (Life Technologies, Carlsbad, CA) was used according to the user's manual. The sequence for scrambled siRNA was 5′-GCAGUGUCUCCACGUACUA-3′. The sequences for FDX2 siRNA were 5′-GCUGCAAUAAAUCGAUAACAC-3′ and 5′-GCUGCCAGAUUGUCUGACAC-3′.

#### RNA Isolation and RT-PCR Analysis

Total RNAs were extracted from cells using TRIzol (Life Technologies), according to the manufacturer's instructions. cDNA was synthesized using RevertAid First Strand cDNA Synthesis Kit (Thermo Fisher, Agawam, MA), according to the manufacturer's protocol. The PCR program used for amplification was as follows: i) 94°C for 5 minutes, ii) 94°C for 45 seconds, iii) 58°C for 45 seconds, iv) 72°C for 30 seconds, and v) 72°C for 10 minutes. From steps ii to iv, the cycle was repeated 22 times for hypoxanthine-guanine phosphoribosyltransferase (HPRT), FASN, and SCD1 and with 28 cycles for FDX2. The primers for HPRT were 5′-CACCGCGTCACATCGCCAGGCCTT-3′ (forward) and 5′-AAACAAGGCCTGGCGATGTGACGC-3′ (reverse). The primers for SCD1 were 5′-TCTACTTGGAAGACGACATTCGCCC-3′ (forward) and 5′-GGTGGTCACGAGCCCATTCATAGAC-3′ (reverse). The primers for FASN were 5′-CCCGCTCTGGTTCATCTGCTCTG-3′ (forward) and 5′-CGAATGGACGATGTCATCAAAGGTG-3′ (reverse). The primers for FDX2 were 5′-GTGATGCATGTCATGGCCGC-3′ (forward) and 5′-ACTCTGCCACTCACTGGGAT-3′ (reverse).

### Plasmid Construction and Cell Line Generation

To generate *Fdx2-KO* murine cells, two single-guide RNA expression vectors, pSpCas9(BB)-2A-Puro-sgFdx1-1 and pSpCas9(BB)-2A-Puro-sgFdx1-2, were used to remove initiation codon in exon 1 and generate frame shift deletions. The generation of single-guide RNA expression vector was performed as described previously.^20^ The sequence for mouse Fdx2–single-guide RNA-1 is 5′-TTTCCAGGCGAGCGGCGTGC-3′, and the sequence for mouse Fdx2–single-guide RNA-2 is 5′-AGGGGCACTCACACGTCCCG-3′. To generate *Fdx2*^*+/–*^ and *Fdx2*^*−/−*^ SCp2 and FL83B cell lines, both guide RNAs were cotransfected into these cells using JetPRIME transfection reagent (Polyplus, Illkirch-Graffenstaden, France). The cells were selected with puromycin, and individual clones were picked, genotyped, sequenced, and confirmed by Western blot analysis. The primers used for genotyping mouse *Fdx2* were 5′-GTCACGTGGCCTATCATG-3′ (forward) and 5′-GACAAACAGGGTGCAGGT-3′ (reverse). The generation of *FDX2*-heterozygous HCT116 and Mia-PaCa2 cell lines has been described previously.^16^

### Western Blot Analysis

Western blot analysis was performed as previously described.^21^ Briefly, cell lysates were collected as indicated, resolved on 8% to 11% SDS-polyacrylamide gels, and transferred to nitrocellulose membrane. After the transfer, the membranes were blocked with phosphate-buffered saline with Tween 20 (PBST) containing 2.5% milk at room temperature for 1 hour, followed by overnight 4^o^C incubation with primary antibodies prepared in 2.5% milk containing PBST. The following day, after 3× washings with PBST, the blots were incubated at room temperature for 1 hour in secondary antibody prepared in PBST containing 2.5% milk. After 3× washings with PBST, the blots were soaked in enhanced chemiluminescence reagents (Thermo Fisher Scientific, Waltham, MA), and then visualized with the BioSpectrum 810 Imaging System (UVP LLC, Upland, CA). Antibody against human/mouse ABCA1 [number PA1-16789; Research Resource Identifier (RRID): AB_2288917] was purchased from Invitrogen Life Technologies (Carlsbad, CA) and Cell Signaling Technology (number 96292; Danvers, MA). Antibodies against SREBP1 (number ab3259; RRID: AB_303650) and SREBP2 (number ab30682; RRID: AB_779079) were purchased from Abcam (Cambridge, MA). FDX2 antibody (HPA043986) was purchased from Atlas Antibodies (Stockholm, Sweden). Horseradish peroxidase–conjugated secondary antibodies against rabbit and mouse IgG were purchased from BioRad (Hercules, CA).

### Nile Red Staining

Nile Red (number ab219403) was purchased from Abcam. A 10 μmol/L stock solution of Nile Red was prepared by dissolving it in acetone. Nile Red was diluted to 2 μg/mL in complete medium and added to the cells for 30 minutes at room temperature. Subsequently, the cells were washed with phosphate-buffered saline and fixed in 4% paraformaldehyde (Sigma Aldrich, St. Louis, MO) for 20 minutes at room temperature. The cells were then counterstained with DAPI, and images were obtained with confocal microscope. To quantitate fluorescence, 1 × 10^5^ cells were stained in triplicate with Nile Red for 20 minutes. Fluorescence was then detected using the SPECTRAmax GEMINI XS (Molecular Devices, San Jose, CA) with an excitation wavelength of 559 nm and an emission wavelength of 635 nm.

### Measurement of Cellular Cholesterol and Triglycerides

Total cholesterol and triglycerides from cells were determined by using Cholesterol/Cholesterol Ester-Glow assay kit (Promega, Madison, WI) and Triglyceride-Glow assay kit (Promega), respectively. Briefly, cells were plated in a 96-well plate. After 4 hours of fasting in serum-free media, the levels of cholesterol and triglycerides were measured according to manufacturer's protocol.

### Histologic Analysis

Wild-type, *Fdxr*^+/−^, *Fdx2*^+/−^, and *Fdx2*^−/−^ mouse tissues were fixed in 10% (w/v) neutral-buffered formalin, processed, and embedded in paraffin blocks. Embedded tissues were sectioned (6 μm thick) and stained with hematoxylin and eosin.

### Untargeted Lipidomic Analysis Using Liquid Chromatography–Tandem Mass Spectrometry

Lipids were extracted and analyzed by reversed-phase liquid chromatography–tandem mass spectrometry at West Coast Metabolomics Center with published methods.^22^ A total of 30 million isogenic control or *FDX2-heterozygous* Mia-PaCa2 cells were harvested and analyzed for lipidomics, as previously described.^23^

### Statistical Analysis

The data were presented as means ± SEM or means ± SD, as indicated. Statistical significance was determined by two-tailed *t*-test. Values of *P* < 0.05 were considered significant. For Kaplan-Meyer survival analysis, log-rank test was performed. Fisher exact test was used for comparison of tumors, adenocarcinomas, and liver steatosis/steatohepatitis from different mice cohorts.

## Results

### Mice Deficient in *Fdx2* Are Viable but Have a Short Lifespan and Are Prone to Steatohepatitis

**Figure 1:**
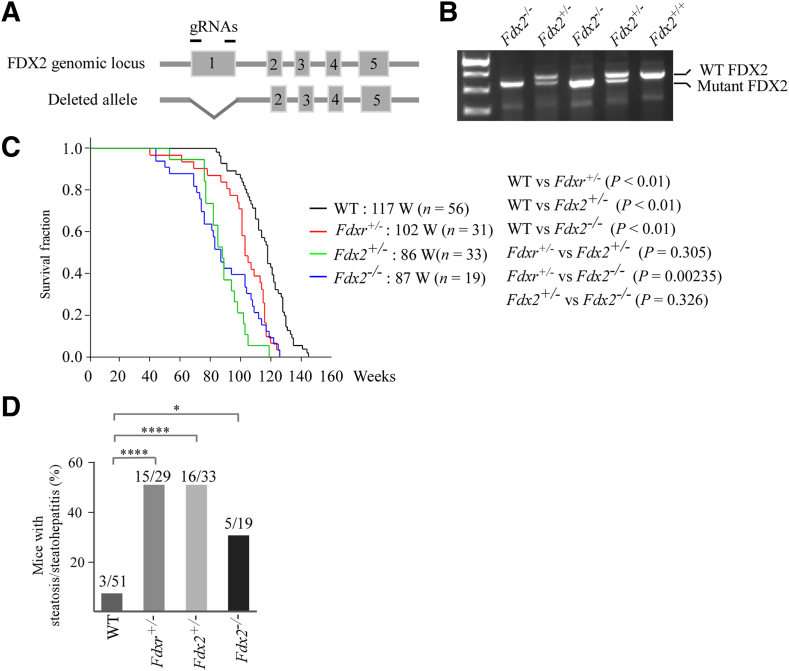
Fdx2 knockout mice are viable, have a shorter lifespan, and are prone to steatohepatitis. **A:** Schematic showing the targeted mouse *Fdx2* gene locus and the location of guide RNAs (gRNAs) used to knock out exon 1. **B:** A representative genotyping PCR gel image showing the mice with *Fdx2*^*+/−*^ and *Fdx2*^*−/−*^ genotype. **C:** Kaplan-Meier survival curves of wild-type (WT), *Fdxr*^*+/−*^, *Fdx2*^*+/−*^, and *Fdx2*^*−/−*^ mice. **D:** The numbers and percentages of WT, *Fdxr*^*+/−*^, *Fdx2*^*+/−*^, and *Fdx2*^*−/−*^ mice with liver steatosis/steatohepatitis. *n* = 56 WT mice (**C**); *n* = 31 *Fdxr*^*+/−*^ mice (**C**); *n* = 33 *Fdx2*^*+/−*^ mice (**C**); *n* = 19 *Fdx2*^*−/−*^ mice (**C**). ∗*P* < 0.05, ∗∗∗∗*P* < 0.0001.

The biochemical and cellular roles of FDX2 in biogenesis of ISCs and sterol hormone biosynthesis have been analyzed since its discovery in 2010.^3^^,^^24^, ^25^, ^26^ Germline deletion of *FDX2* in humans leads to mitochondriopathy,^27^^,^^28^ which is linked to its electron transfer function within the mitochondria. However, other physiological roles of FDX2 have not been explored. In this regard, an *Fdx2*-deficient mouse model, in which the exon 1 in the *Fdx2* gene was ablated by clustered regularly interspaced short palindromic repeats–CRISPR-associated protein 9 (CRISPR-Cas9**)** (Figure 1, A and B), was obtained from the RIKEN BioResource Research Center in Japan. On breeding *Fdx2*^*+/–*^ mice, *Fdx2*^*−/−*^ mice were found to be viable and indistinguishable from their wild-type littermates, which was in sharp contrast from the embryonic lethality observed in *Fdxr-* and *Fdx1*-deficient mice.^16^^,^^17^ These data suggest that the pathways regulated by Fdx2 are not required for mammalian embryonic development and reproduction. These findings also indicate that although Fdxr, Fdx1, and Fdx2 are closely related, they possess both distinct and overlapping functions.

To explore the physiological significance of Fdx2, a cohort of *Fdx2*^*+/–*^ and *Fdx2*^*−/−*^ mice were generated and monitored throughout their lifespan for their susceptibility to cancer and other pathologic alterations. To minimize the number of animals, the wild-type mice were adapted from previous studies.^16^^,^^29^ Additionally, *Fdxr*^*+/–*^ mice from a previous study^16^ were used for comparison because Fdx2 is a substrate of Fdxr. All the mice used here were of C57BL/6 background and raised in the same animal facility. The median survival time was 86 weeks for the *Fdx2*^*+/−*^ mice (*n* = 33) and 87 weeks for the *Fdx2*^*−/−*^ mice (*n* = 19), and there was no statistical significance in the median survival between *Fdx2*^*+/−*^ and *Fdx2*^*−/−*^ mice (*P* = 0.326 by log-rank test). Interestingly, the median survival for *Fdx2*^*+/−*^ and *Fdx2*^*−/−*^ mice was significantly shorter than that for wild-type mice (117 weeks; *n* = 56) or for the *Fdxr*^*+/−*^ mice (102 weeks; *n* = 31) (Figure 1C and Supplemental Tables S1–S4).

Next, to examine the pathologic abnormality mediated by Fdx2 deficiency, gross necropsy and histologic analyses were performed with the major organs from *Fdx2*-deficient mice. Indeed, 15 of 33 *Fdx2*^*+/−*^ mice and 9 of 19 *Fdx2*^*−/−*^ mice developed spontaneous tumors (Table 1, Supplemental Figure S1A, and Supplemental Tables S3 and S4). Statistical analyses indicated that the tumor incidence was significantly higher in both *Fdx2*^*+/−*^ and *Fdx2*^*−/−*^ mice than that in WT mice (WT versus *Fdx2*^*+/−*^: *P* = 0.03 by Fisher exact test; WT versus *Fdx2*^*−/−*^: *P* = 0.04 by Fisher exact test) (Table 1 and Supplemental Tables S1–S4). Interestingly, when comparing the tumor incidence of *Fdx2*^*+/−*^ and *Fdx2*^*−/−*^ mice with that of *Fdxr*^*+/−*^ mice, the tumor incidence was significantly lower in *Fdx2*^*+/−*^ or *Fdx2*^*−/−*^ mice than in *Fdxr*^*+/−*^ mice (*Fdxr*^*+/−*^ versus *Fdx2*^*+/−*^: *P* = 0.0004 by Fisher exact test; *Fdxr*^*+/−*^ versus *Fdx2*^*−/−*^: *P* = 0.002 by Fisher exact test) (Table 1 and Supplemental Tables S1–S4).

**Table 1:** Tumor Spectra in WT, *Fdxr*^+/–^, *Fdx2*^+/–^, and *Fdx2*^−/−^ Mice

Tumor	WT mice (*n* = 51)	*Fdxr*^+/–^ mice (*n* = 29)	*Fdx2*^+/–^ mice (*n* = 33)	*Fdx2*^−/−^ mice (*n* = 19)
Lymphoma	11	19	9	5
Sarcoma	1	7	1	1
Adenocarcinoma	0	3	3	2
Hepatocellular carcinoma	0	2	2	0
Granulosa cell tumor	0	0	1	0
Adenoma	0	0	1	0
Hematoma	0	0	1	0
Lipoma	0	0	0	1
Tumor penetrance	11/51	26/29	15/33	9/19

Wild-type (WT) mice were from published studies (Zhang et al^16^ and Yang et al^29^). *Fdxr*^*+/–*^ mice were from published studies (Zhang et al^16^ and Zhang et al^30^). Found dead mice were excluded from tumor study.

*Fdx2*-deficient mice were more obese than their WT littermates (Supplemental Figure S1, B and C), suggesting that Fdx2 deficiency alters lipogenesis. In addition to spontaneous tumors, histologic analyses indicated that similar to *Fdxr*^*+/−*^ mice, *Fdx2*^*+/−*^ and *Fdx2*^*−/−*^ mice exhibited significantly higher incidence of steatosis/steatohepatitis compared with the WT mice (Figure 1D, Supplemental Figure S1D, and Supplemental Tables S1–S4). The findings were also consistent with the ones from the International Mouse Phenotyping Consortium (*https://www.mousephenotype.org*, last accessed October 30, 2024), indicating that *Fdx2*^*+/−*^ mice weighted more than the WT mice and had liver abnormalities. As Fdx2 is closely related to Fdxr and Fdx1, the levels of Fdxr and Fdx1 proteins were also measured in the livers from male and female WT and *Fdx2*^*−/−*^ mice. Indeed, the levels of Fdxr and Fdx1 proteins were comparable in WT and *Fdx2*^*−/−*^ livers (Supplemental Figure S1E), indicating that Fdx2 does not regulate the expression of Fdxr or Fdx1 but rather transduces the Fdxr signals. Together, these data reinforce the critical role of the Fdxr-Fdx1/2 pathway in regulating lipid metabolism.^15^, ^16^, ^17^

### FDX2 Deficiency Alters Lipid Metabolism, Potentially via ABCA1-SREBP1/2

**Figure 2:**
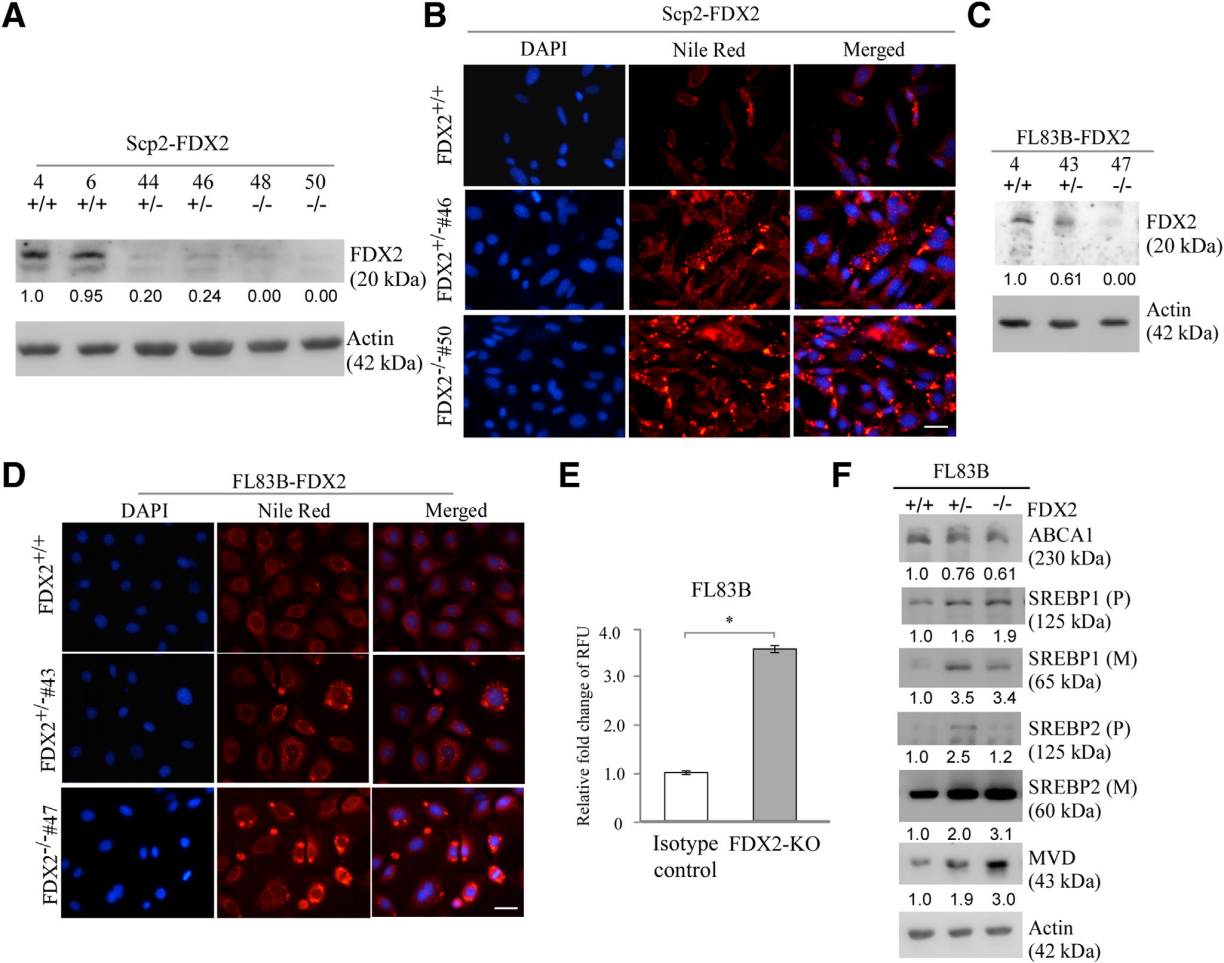
Lipid metabolism is altered in murine cells lacking FDX2, potentially via ATP-binding cassette transporter A1 (ABCA1)–sterol regulatory element-binding proteins 1/2 (SREBP1/2) pathways. **A:** The levels of Fdx2 and actin were measured in two sets of isogenic control, *Fdx2*^*+/−*^, and *Fdx2*^*−/−*^ SCp2 cells. The relative fold of protein levels was shown below each lane. Actin was used as internal control. **B:** Nile Red staining was used to visualize lipid droplets in isogenic control, *Fdx2*^*+/−*^, and *Fdx2*^*−/−*^ SCp2 cells cultured in serum-free media for 4 hours. Nuclei were stained with DAPI. **C:** The levels of Fdx2 and actin were measured in isogenic control, *Fdx2*^*+/−*^, and *Fdx2*^*−/−*^ FL83B cells. The relative fold of protein levels was shown below each lane. Actin was used as internal control. **D:** Isogenic control, *Fdx2*^*+/−*^ (clone number 43), and *Fdx2*^*−/−*^ (clone number 47) FL83B cells were starved in serum-free media for 4 hours, followed by Nile Red staining to visualize lipid droplets. DAPI was used to stain nuclei. **E:** A total of 1 × 10^5^ isogenic control and *Fdx2*^*−/−*^ FL83B cells were stained with Nile Red for 20 minutes, followed by measurement of fluorescence units. Data are presented as relative fold change of fluorescence units. **F:** The levels of ABCA1, SREBP1/2, mevalonate diphosphate decarboxylase (MVD), and actin were measured in isogenic control, *Fdx2*^*+/−*^, and *Fdx2*^*−/−*^ FL83B cells starved in serum-free media for 4 hours. The relative fold of protein levels was shown below each lane. Actin was used as internal control. ∗*P* < 0.05 (*t*-test). Scale bar = 5 μm (**B** and **D**). KO, knockout; M, mature protein; p, precursor; RFU, relative fluorescence unit.

Given that the *Fdx2*-deficient mice were obese and had a high incidences of steatosis, the authors postulated that FDX2 plays a role in lipid metabolism. To test this, CRISPR-Cas9 was used to knock out *Fdx2* in mouse SCp2 mammary epithelial cells with the same pair of guide RNAs to delete exon 1 in the *Fdx2* gene locus. Multiple *Fdx2*^*+/−*^ and *Fdx2*^*−/−*^ SCp2 cell clones were generated and sequence confirmed. Western blot analysis showed that Fdx2 protein was undetectable in two *Fdx2*^*−/−*^ clones and much lower in two *Fdx2*^*+/−*^ clones compared with two isogenic control clones (Figure 2A). To determine the role of FDX2 in lipid metabolism, these cells were stained with Nile Red (9-diethylamino-5H-benzo[a]phenoxazine-5-one), which is known to stain lipid droplets. Atrong cytoplasmic Nile Red staining in *Fdx2*^*+/−*^ and *Fdx2*^*−/−*^ SCp2 cells was observed compared with isogenic control cells (Figure 2B), suggesting that Fdx2 deficiency led to altered lipid metabolism and accumulation of lipid droplets. To corroborate the above findings, FL83B, a murine hepatocyte cell line, was also used to ablate *Fdx2* using CRISPR-Cas9. As in SCp2 cells, *Fdx2*^*+/−*^ and *Fdx2*^*−/−*^ clones were obtained. Immunoblotting showed that Fdx2 protein was undetectable in *Fdx2*-null FL83B cells and markedly reduced in *Fdx2*^*+/−*^ FL83B cells (Figure 2C). Similarly, Nile Red staining showed that lipid was accumulated in *Fdx2*^*+/−*^ FL83B cells and further accumulated in *Fdx2*^*−/−*^ FL83B cells compared with that in isogenic control cells (Figure 2D), consistent with the observations in SCp2 cells. To verify this, the Nile Red fluorescence was measured, and relative fluorescence units were markedly increased in FDX2-KO FL83B cells compared with those in the isogenic control FL83B cells (Figure 2E).

FDXR regulates lipid metabolism via the ABCA1-SREBP1/2 pathway.^15^ As a substrate of FDXR, it is likely that FDX2 may recruit ABCA1-SREBP1/2 to regulate lipid metabolism. To test this, isogenic control, *Fdx2*^*+/−*^, and *Fdx2*^*−/−*^ FL83B cells were cultured in serum-free medium to mimic cholesterol depletion. Fdx2 deficiency decreased the level of ABCA1, the cholesterol efflux pump,^31^ in *Fdx2*^*+/−*^ and *Fdx2*^*−/−*^ FL83B cells compared with that in isogenic control cells (Figure 2F), in accordance with the observation in *FDXR*-deficient cells.^15^^,^^32^ Moreover, SREBP1, a SREBP family member involved in fatty acid and TAG synthesis,^33^^,^^34^ was increased by *Fdx2* deficiency (Figure 2F). Furthermore, the mature form of SREBP2, which functions as a nuclear transcription factor and is associated with low intracellular cholesterol and ABCA1 levels (Moon et al^32^), was increased in *Fdx2*^*+/−*^ and *Fdx2*^*−/−*^ FL83B cells (Figure 2F). To examine whether the mature nuclear form of SREBP2 is transcriptionally active, the level of mevalonate diphosphate decarboxylase was measured, which catalyzes the decarboxylation of mevalonate 5-diphosphate into isopentenyl diphosphate, the final step in the mevalonate pathway.^35^^,^^36^ Mevalonate diphosphate decarboxylase is a target of SREBP1/2^37^ and plays a key role in cholesterol biosynthesis. Indeed, the level of mevalonate diphosphate decarboxylase protein was increased in *Fdx2*-deficient cells (Figure 2F).

**Figure 3:**
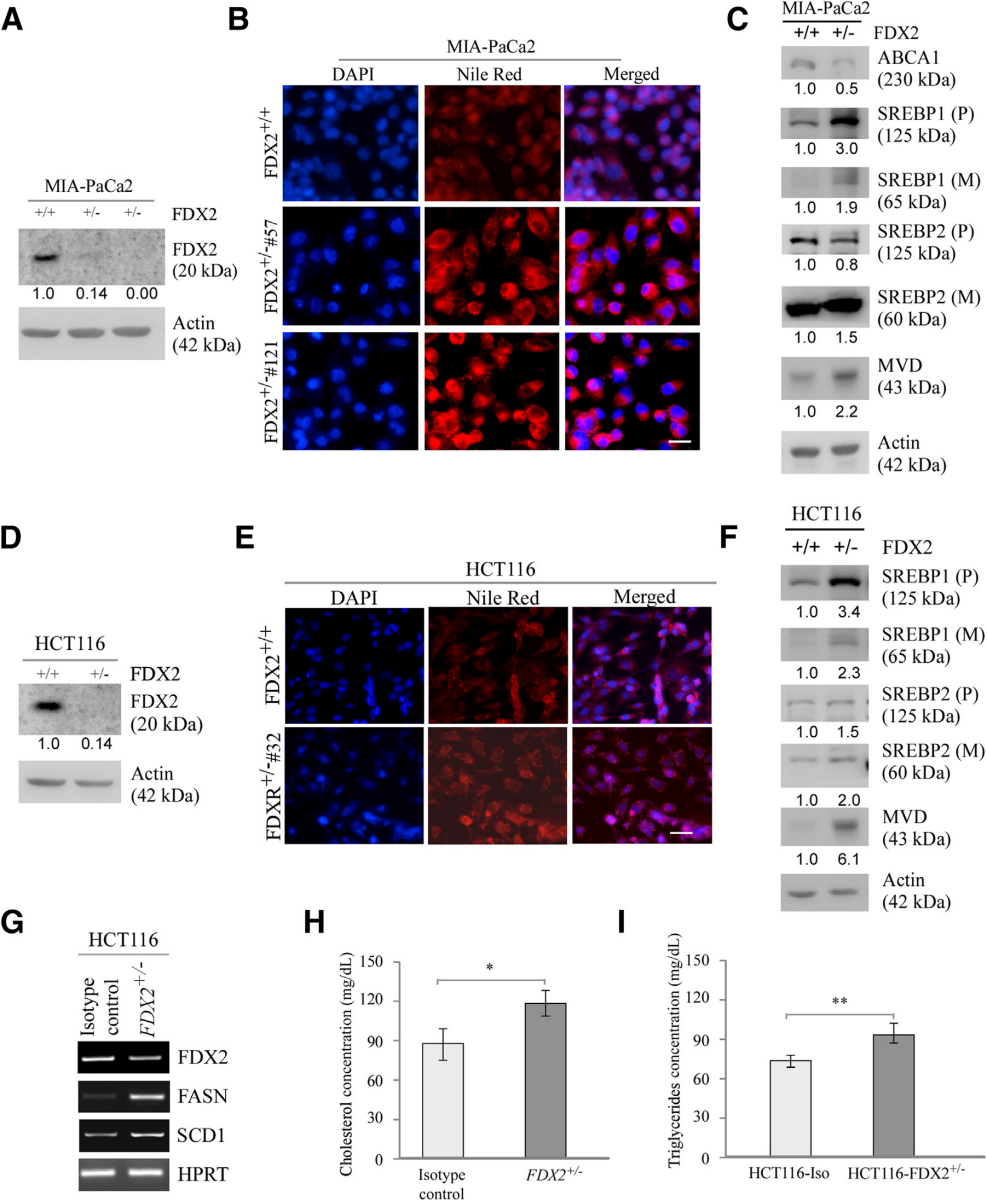
Lipid metabolism is altered in human cells lacking FDX2, potentially via ATP-binding cassette transporter A1 (ABCA1)–sterol regulatory element-binding proteins 1/2 (SREBP1/2) pathways. **A** and **D:** Immunoblotting was used to measure the levels of FDX2 and actin proteins in isogenic control and *FDX2*^*+/−*^ Mia-PACA2 (**A**) or HCT116 (**D**) cells. The relative fold of protein levels was shown below each lane. Actin was used as internal control. **B** and **E:** Nile Red staining was used to visualize lipid droplets in isogenic control and *FDX2*^*+/−*^ Mia-PACA2 (**B**) or HCT116 (**E**) cells starved in serum-free media for 4 hours. Nuclei were stained with DAPI. **C** and **F:** Immunoblotting was used to measure the levels of ABCA1, SREBP1/2, mevalonate diphosphate decarboxylase (MVD), and actin in isogenic control and *FDX2*^*+/−*^ Mia-PACA2 (**C**) or HCT116 (**F**) cells starved in serum-free media for 4 hours. The relative fold of protein levels was shown below each lane. Actin was used as internal control. **G:** The levels of FDX2, fatty acid synthase (FASN), stearoyl-CoA desaturase 1 (SCD1), and hypoxanthine-guanine phosphoribosyltransferase (HPRT) mRNA were measured in isogenic control and *FDX2*^*+/−*^ FL83B cells. **H:** Cholesterol/Cholesterol Ester-Glo assay kit was used to measure the level of total cholesterol in isogenic control and *FDX2*^*+/−*^ HCT116 cells that were starved in serum-free media for 4 hours. **I:** Triglyceride-Glo assay kit was used to measure the level of total cholesterol in isogenic control and *FDX2*^*+/−*^ HCT116 cells that were starved in serum-free media for 4 hours. Data represent the means ± SD (**H** and **I**). ∗*P* < 0.05, ∗∗*P* < 0.01. Scale bar = 5 μm (**B** and **E**). M, mature protein; p, precursor.

Although FDX2 is highly conserved across species,^1^ it is not clear whether its physiological function in lipid metabolism is conserved. To test this, human cell lines MIA-PaCa2 and HCT116 were used to generate *FDX2*-deficient clones by CRISPR-Cas9.^16^ Although *FDX2*^*+/−*^ clones were readily generated, no single FDX2-null clone was generated in human MIA-PaCa2 and HCT116 cells (Figure 3, A and D), suggesting that the requirement of FDX2 for cell survival is different between humans and murine animals. Nevertheless, the level of FDX2 protein was markedly decreased in *FDX2*^*+/−*^ MIA-PaCa2 and HCT116 cells compared with that in isogenic control cells (Figure 3, A and D). Additionally, FDX2 deficiency had no effect on the expression of FDXR and FDX1 in both Mia-PaCa2 and HCT116 cells (Supplemental Figure S2), consistent with the data obtained in mouse tissues (Supplemental Figure S1E). Whether FDX2 deficiency had any effect on the accumulation of lipid droplets by performing Nile Red staining was examined next. FDX2 deficiency promoted lipid droplet accumulation in the cytoplasm in both Mia-PACA2 and HCT116 cells compared with their respective isogenic control cells (Figure 3, B and E). Moreover, FDX2 deficiency led to maturation and activation of SREBP1/2 proteins and induction of mevalonate diphosphate decarboxylase in Mia-PaCa2 and HCT116 cells (Figure 3, C and F). In line with this, the transcripts of several SREBP1/2 targets, including FASN and SCD1, were increased in *FDX2*^*+/−*^ HCT116 cells compared with isogenic control HCT116 cells (Figure 3G). Furthermore, the levels of intracellular TAGs and cholesterol were measured in isogenic control and *FDX2*^*+/−*^ HCT116 cells. *FDX2* deficiency led to a marked increase in the levels of cholesterol and TAGs (Figure 3, H and I).

**Figure 4:**
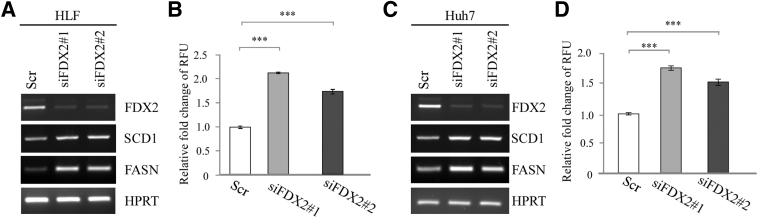
Knockdown of FDX2 alters lipid genes as well as lipid droplets in HLF and Huh7 cells. HLF (**A**) and Huh7 (**C**) cells were transiently transfected with a scrambled (Scr) siRNA or siRNAs against FDX2 (siFDX2#1 and siFDX2#2) for 3 days, followed by RT-PCR analysis to measure the level of FDX2, fatty acid synthase (FASN), stearoyl-CoA desaturase 1 (SCD1), and hypoxanthine-guanine phosphoribosyltransferase (HPRT) transcripts. HLF (**B**) and Huh7 (**D**) were transiently transfected with a scrambled siRNA or siRNAs against FDX2 for 3 days, stained with Nile Red for 20 minutes, followed by measurement of fluorescent intensity. ∗∗∗*P* < 0.001 (*t*-test). RFU, relative fluorescence unit.

To further verify that FDX2 deficiency led to altered lipid accumulation, two more human hepatocellular carcinoma cell lines, HLF and Huh7, were used. Briefly, HLF and Huh7 were transiently transfected with a scrambled siRNA or siRNAs against FDX2, followed by RT-PCR analysis and measurement of Nile Red fluorescence. Knockdown of FDX2 led to increased levels of SCD1 and FASN transcripts in both HLF and Huh7 cells (Figure 4, A and C). Moreover, the relative fluorescence units were markedly increased by FDX2 knockdown in HLF and Huh7 cells (Figure 4, B and D).

### Overexpression of FDX2 Decreases Lipid Accumulation

**Figure 5:**
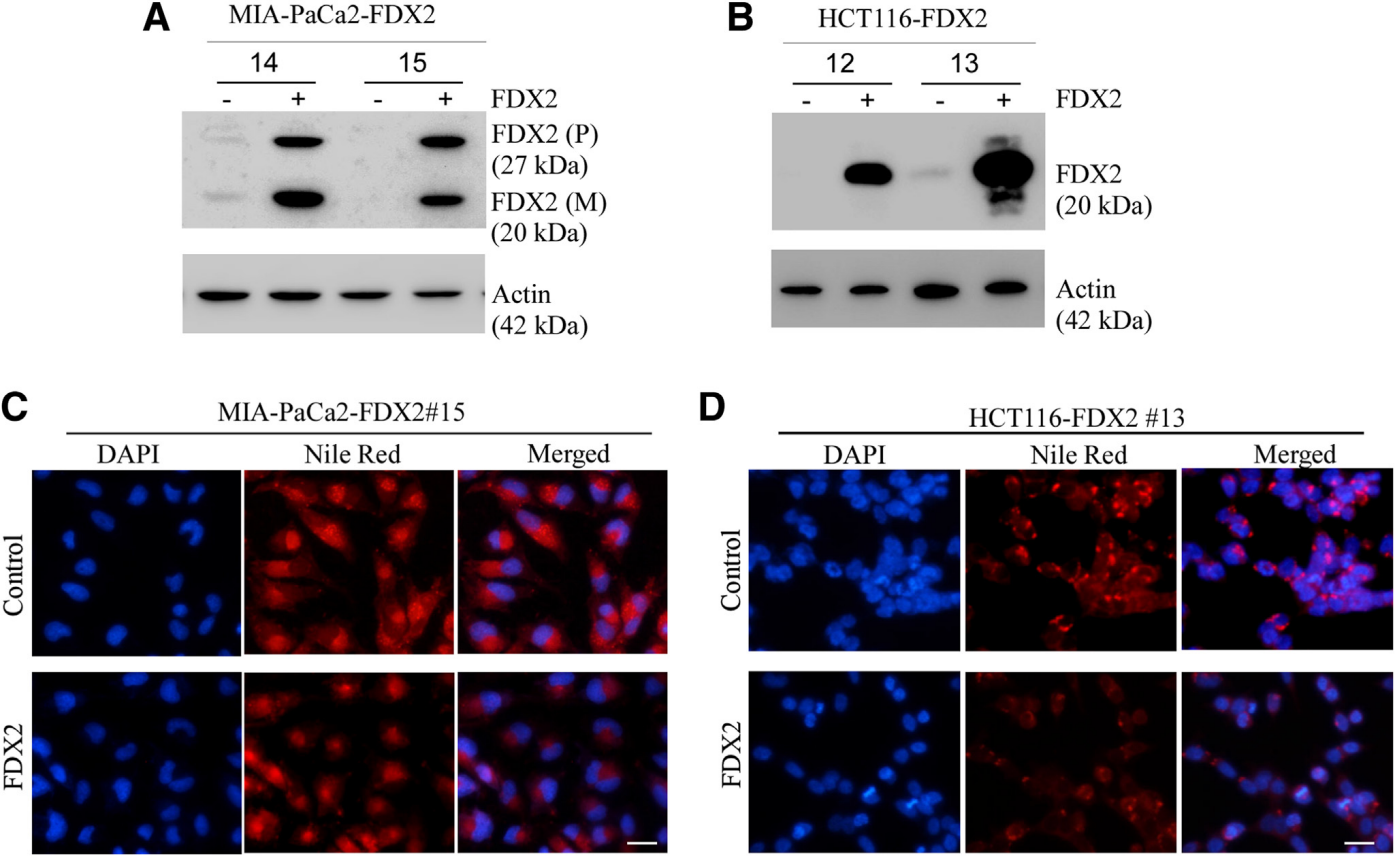
Ectopic expression of FDX2 decreases lipid droplet accumulation. Immunoblotting was used to measure the levels of FDX2 and actin proteins in two FDX2-expressing Mia-PACA2 (**A**) or HCT116 (**B**) clones with or without induction with doxycycline. Nile Red staining was used to visualize lipid droplets after FDX2 induction (24 hours with doxycycline) in Mia-PACA2 (**C**) or HCT116 (**D**) cells starved in serum-free media for 4 hours. Nuclei were stained using DAPI. Scale bar = 5 μm (**C** and **D**). M, mature protein; p, precursor.

To further confirm that FDX2 plays a role in lipid metabolism, multiple MIA-PaCa2 and HCT116 cell lines were generated in which FDX2 could be inducibly expressed under the control of tetracycline (doxycycline)–inducible promoter. On induction with doxycycline, FDX2 protein was highly expressed in Mia-PaCa2 and HCT116 cells (Figure 5, A and B). The slow migrating FDX2 is likely to be the uncleaved form (Figure 5A) because the mitochondrial signal peptide in FDX2 is cleaved upon translocated inside mitochondria. Next, Nile Red staining was performed to examine accumulation of cytoplasmic lipid droplets with cells uninduced or induced to express FDX2. As opposed to the increased lipid droplet accumulation in FDX2-deficient cells, overexpression of FDX2 led to a moderate decrease in lipid droplet accumulation in Mia-PaCa2 cells and a robust decrease in lipid droplets in HCT116 cells (Figure 5, C and D).

### Lipid Profile Alterations in FDX2-Deficienct Cells

**Figure 6:**
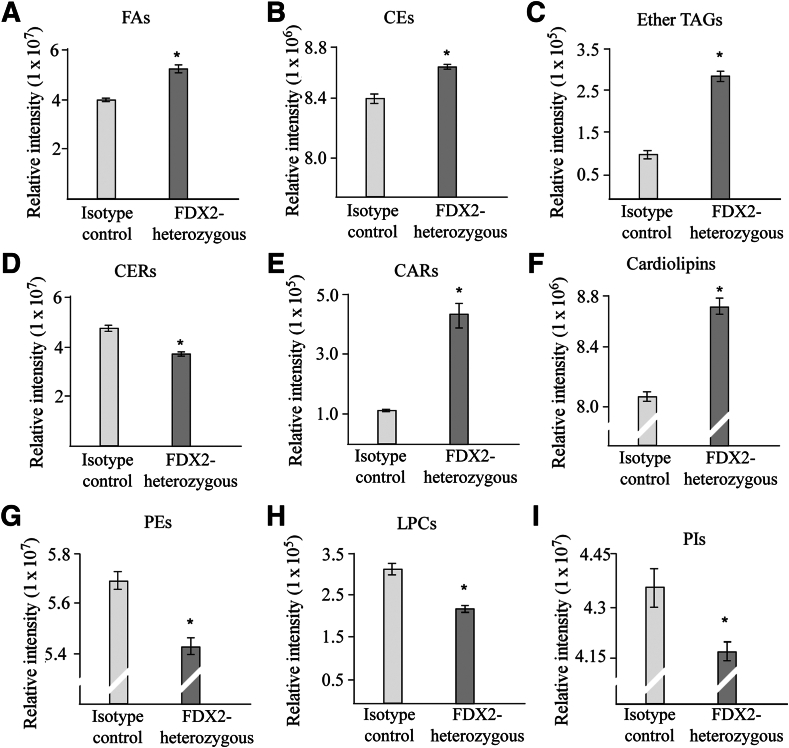
FDX2 deficiency leads to alterations in the lipid profile. Isogenic control and FDX2-heterozygous HCT116 cells were used for lipidomic analysis by liquid chromatography–tandem mass spectrometry. The relative abundance of a lipid was calculated, and statistical significance was determined using *t*-test. **A:** Fatty acids (FAs). **B:** Cholesterol esters (CEs). **C:** Ether triacylglycerides (Ether TAGs). **D:** Ceramides (CERs). **E:** Acylcarnidines (CARs). **F:** Cardiolipins. **G:** Phosphatidylethanolamines (PEs). **H:** Lysophosphatidylcholines (LPCs). **I:** Phosphatidylinositols (PIs). Data are presented as means ± SEM (**A**–**I**). ∗*P* < 0.05.

To examine the specific classes of lipids altered in FDX2-deficient cells, untargeted lipidomic analysis was performed with isogenic control and *FDX2*^*+/−*^ HCT116 cells. FDX2 deficiency increased the content of fatty acids, cholesterol, and ether TAGs (Figure 6, A–C), which is consistent with finding that loss of FDX2 leads to increased lipid droplets as well as cholesterol level (Figures 2, 3, and 4). Also, FDX2 deficiency decreased the content of ceramides but increased the content of acylcarnitines and cardiolipins (Figure 6, D–F). Furthermore, FDX2 deficiency decreased the content of various phospholipids, including phosphatidylethanolamines, lysophosphatidylcholines, and phosphatidylinositol (Figure 6, G–I). Taken together, the data indicate that FDX2 deficiency alters lipid metabolism, leading to a pronounced alteration of lipid profiles.

## Discussion

FDX2, a member of the ferredoxin family, is recognized for its essential function in mitochondrial biology, particularly in the assembly of ISCs crucial for various cellular processes.^1^ This study delved into its physiological role by using the *Fdx2*-deficient mouse model. It showed that mice deficient in *Fdx2* have a short lifespan and are prone to spontaneous tumors and steatohepatitis. However, unlike Fdxr and Fdx1, Fdx2 is not required for mammalian embryonic development. Furthermore, FDX2 deficiency led to altered lipid metabolism by modulating the ABCA1-SREBP1/2 pathways. This study contributes to the growing understanding of the ferredoxin family's diverse roles in tumorigenesis and lipid metabolism.

Previous studies showed a crosstalk between iron and lipid pathways.^38^ For example, increased hepatic iron is correlated with increased cholesterol content.^39^ However, the underlying mechanism is not clear. This study showed that loss of FDX2, a critical player in ISC biogenesis, leads to altered lipid accumulation in various human and mouse cells (Figures 2, 3, and 4). Consistent with this, ectopic expression of FDX2 inhibited lipid accumulation (Figure 5). Furthermore, lipidomic analysis showed that loss of FDX2 led to increased levels of various lipids, including fatty acids, cholesterol, ether triglycerides, and carnitines (Figure 6). Studies exploring the underlying mechanism indicated that loss of FDX2 led to decreased expression of ABCA1, which, in turn, led to increased expression of SREBP1/2 (Figures 2F, and 3, C and F). SREBP1/2 are membrane-bound transcription factors that directly activate the expression of >30 genes dedicated to the synthesis and uptake of cholesterol, fatty acids, triglycerides, and phospholipids.^40^, ^41^, ^42^ By contrast, ABCA1 mediates cholesterol and phospholipid efflux and, thereby, decreases lipid accumulation.^43^^,^^44^ Together, the current data suggest that FDX2 modulates lipid metabolism, possibly through the regulation of ABCA1 and the subsequent impact on SREBP1/2 activation. This connection highlights a novel aspect of FDX2 function, providing a mechanistic link between mitochondrial ISC assembly and lipid homeostasis. Nevertheless, several questions remain to be addressed. First, it would be interesting to determine how FDX2 modulates expression of ABCA1 and SREBP1/2. Second, in addition to cholesterol, loss of FDX2 leads to increase in several other lipids, such as fatty acids and ether TAG (Figure 6). It would be interesting to determine whether FDX2 modulates these lipid pathways via the ABCA1-SREBP1/2 pathway.

FDX1 deficiency leads to embryonic lethality in mice.^17^ Here, Fdx2 did not affect embryonic development, suggesting that FDX1 and FDX2 have distinct roles in development. While *Fdx2*-null mouse cell lines and mice could be generated, FDX2-KO human cells could not (Figures 1, 2, and 3), consistent with other reports.^3^ One possibility is that the activity of mouse Fdx2 can be compensated by mouse Fdx1, whereas human FDX1 cannot compensate for human FDX2. The second possibility is that because Fdx1 is highly expressed in the adrenal glands,^24^ lack of Fdx1 inhibits sterol hormone synthesis, leading to aborted embryogenesis. The third possibility is that the critical role of Fdx2 in ISC biogenesis can be performed by another unknown member of the FDX family in mice. These and other possibilities merit further investigation.

The current study showed that *Fdx2*-deficient mice have a short lifespan and are prone to spontaneous tumors and steatohepatitis (Figure 1). Although the exact mechanism by which Fdx2 deficiency leads to these pathologic abnormalities is unclear, it is plausible to speculate that the spontaneous tumors and lipid alterations mediated by Fdx2 deficiency can form a vicious cycle that exacerbates both conditions, thereby leading to the reduced lifespan. Altered lipid metabolism interacts with the tumor microenvironment to promote tumor progression and resistance to therapy.^45^^,^^46^ As a result, the SREBP1/2 pathway is frequently activated in various cancers, including liver and breast cancers.^47^ On the other hand, tumors frequently modify lipid metabolism to satisfy their elevated energy needs.^48^ Cancer cells may enhance lipid biosynthesis pathways, resulting in lipid droplet accumulation and altered lipid profiles. This lipid dysregulation supports tumor growth and supplies essential structural components for rapidly dividing cells. Nevertheless, further studies are needed to clarify the mechanisms underlying these interactions mediated by Fdx2 deficiency and to assess whether Fdx2 can be targeted for treatment of tumors and associated pathologies.

In conclusion, the current study unveils an essential role of FDX2 in tumor suppression and lipid homeostasis, emphasizing its impact on overall mammalian health. The identified pathways and lipidomic alterations provide a foundation for further investigations into the intricate connections between mitochondrial function, ISC assembly, and lipid metabolism. As our understanding of these relationships deepens, potential implications for therapeutic interventions in conditions associated with aberrant lipid metabolism may emerge.

## Disclosure Statement

None declared.

## Supplemental Data

Supplemental Figure S1 **A:** Representative images of hematoxylin and eosin (H&E)–stained tumors from *Fdx2*^*−/−*^ mice. **B:** Representative images of four *Fdx2*^*+/–*^ mice along with their respective wild-type (WT) littermates. **C:** Representative images of three *Fdx2*^*−/−*^ mice along with their respective WT littermates. **D:** Representative images of H&E-stained wild-type and *Fdx2*^*−/−*^ liver tissues from female (F) and male (M) mice. **E:** The levels of FDX reductase, FDX1, and actin proteins were measured in wild-type and *Fdx2*^*−/−*^ liver tissues from female and male mice. The relative fold of protein levels was shown below each lane. Actin was used as internal control. Scale bar = 5 μm (**A** and **D**).

Supplemental Figure S2 The levels of FDX reductase (FDXR), FDX1, and actin proteins were measured in isogenic control (Iso. Ctrl) and FDX2^+/–^ Mia-PaCa2 (**A**) and HCT116 (**B**) cells. GAPDH, glyceraldehyde-3-phosphate dehydrogenase.
