# Supplementary figures and images for: Ferredoxin 2 Is Critical for Tumor Suppression and Lipid Homeostasis but Dispensable for Embryonic Development
Source: Am J Pathol. 2024 Dec 26;195(4):705–16. doi: 10.1016/j.ajpath.2024.12.002 (PMC13169309; doi:10.1016/j.ajpath.2024.12.002)

Supplementary Figure S1

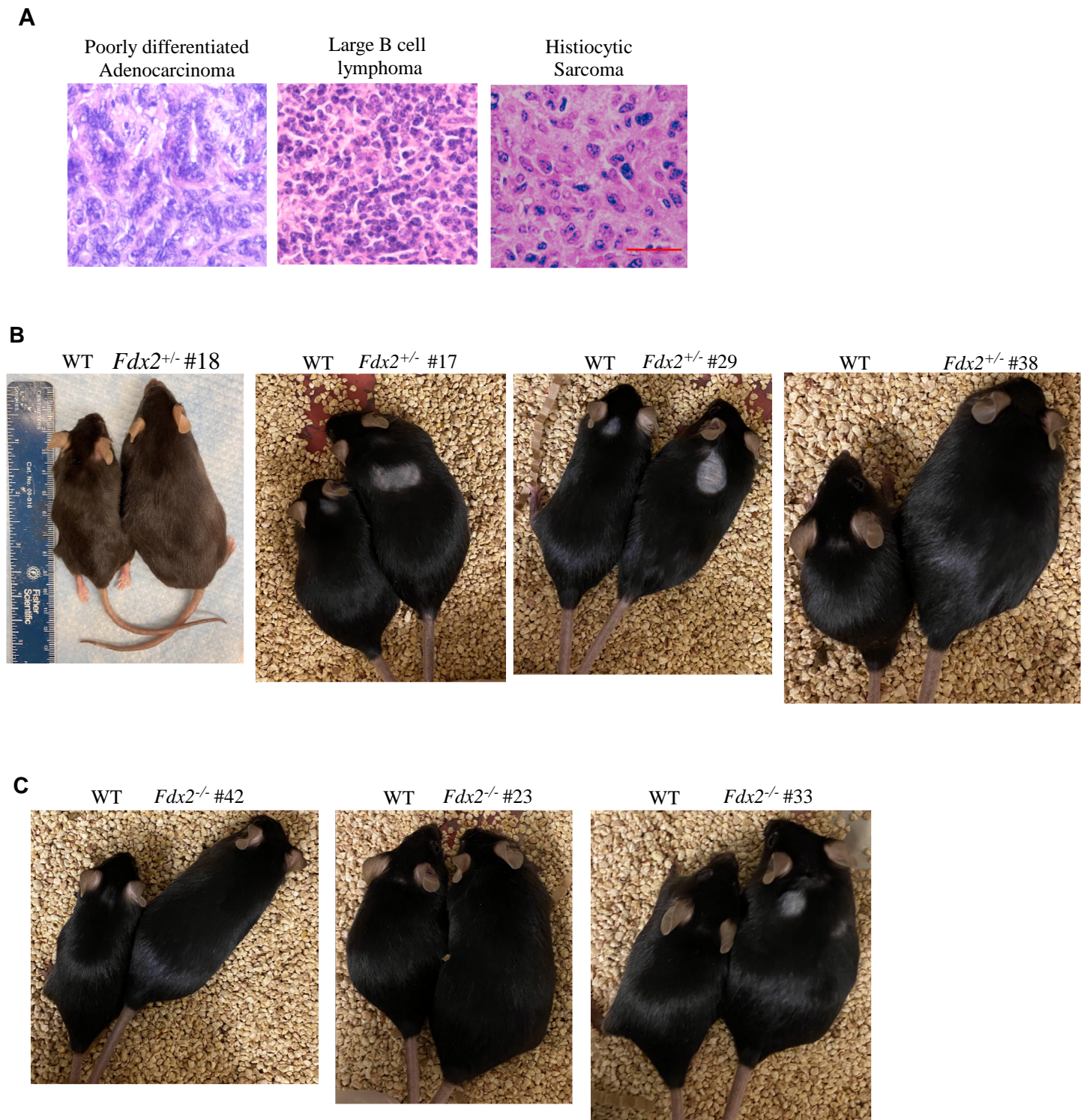

**D**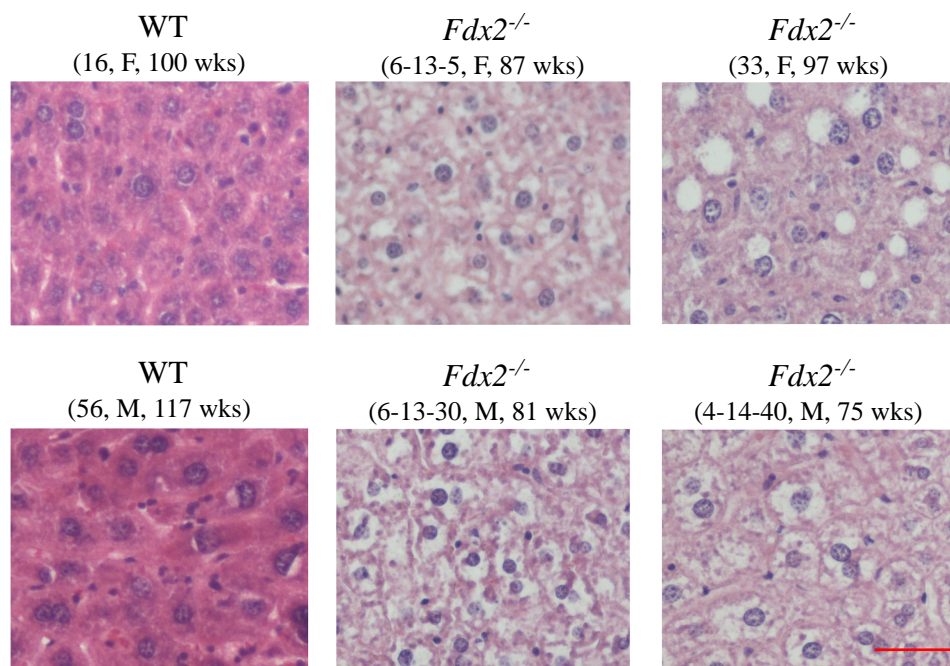**E**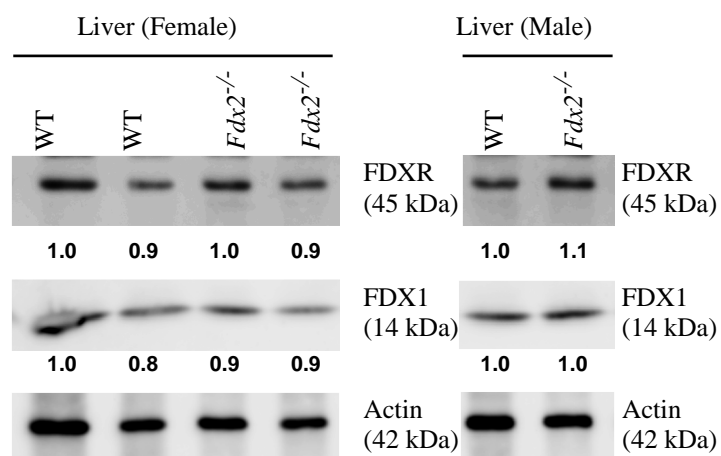

Supplement: Supplemental Figure S1 — A: Representative images of hematoxylin and eosin (H&E)–stained tumors from Fdx2−/− mice. B: Representative images of four Fdx2+/– mice along with their respective wild-type (WT) littermates. C: Representative images of three Fdx2−/− mice along with their respective WT littermates. D: Representative images of H&E-stained wild-type and Fdx2−/− liver tissues from female (F) and male (M) mice. E: The levels of FDX reductase, FDX1, and actin proteins were measured in wild-type and Fdx2−/− liver tissues from female and male mice. The relative fold of protein levels was shown below each lane. Actin was used as internal control. Scale bar = 5 μm (A and D). [file mmc1.pdf]

## Supplementary Figure S2

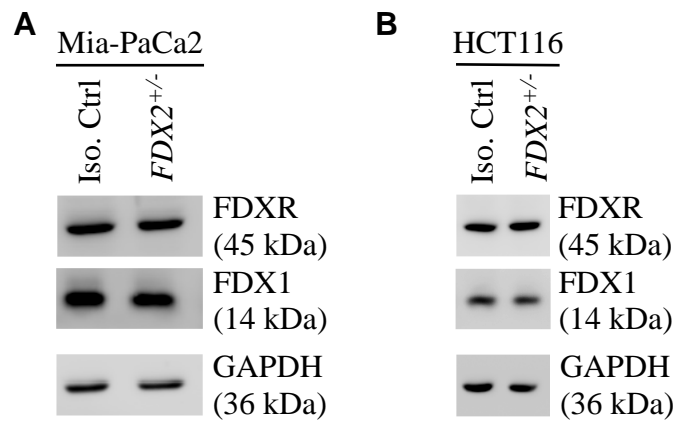

Supplement: Supplemental Figure S2 — The levels of FDX reductase (FDXR), FDX1, and actin proteins were measured in isogenic control (Iso. Ctrl) and FDX2+/– Mia-PaCa2 (A) and HCT116 (B) cells. GAPDH, glyceraldehyde-3-phosphate dehydrogenase. [file mmc2.pdf]
