# Supplementary material for: Ferredoxin 2 Is Critical for Tumor Suppression and Lipid Homeostasis but Dispensable for Embryonic Development
Source: Am J Pathol. 2024 Dec 26;195(4):705–16. doi: 10.1016/j.ajpath.2024.12.002 (PMC13169309; doi:10.1016/j.ajpath.2024.12.002)
Supplement: Supplemental Table S1 [file mmc3.docx]

**Supplemental Table S1.** Wild type (WT) mice (n=56) - survival time, tumor spectrum, steatosis, inflammation, and other abnormalities.

| **ID** | **Sex** | **Survival (Wks)** | **Tumor** | **Steatosis** | **Inflammation** | **Other abnormalities** |
| --- | --- | --- | --- | --- | --- | --- |
| 5 | F | 134 | No | No | No | No |
| 7 | F | 117 | No | No | No | No |
| 16 | F | 100 | No | No | No | No |
| 22 | F | 109 | No | No | No | No |
| 25 | F | 109 | No | No | No | No |
| 44 | F | 90 | No | No | No | No |
| 55 | F | 104 | T-LBL | No | No | No |
| 64 | F | 120 | No | No | No | No |
| 2 | M | 127 | No | No | No | No |
| 3 | M | 117 | No | No | No | No |
| 12 | M | 127 | No | No | Liver | No |
| 13 | M | 127 | No | No | No | No |
| 20 | M | 122 | No | No | No | No |
| 23 | M | 122 | No | No | No | No |
| 26 | M | 127 | No | No | Liver/Salivary gland | No |
| 34 | M | 124 | DLBCL | No | No | No |
| 37 | M | 134 | No | No | No | No |
| 62 | M | 128 | No | No | No | No |
| 45 | M | 133 | No | No | No | No |
| 49 | M | 117 | No | No | No | No |
| 50 | M | 113 | T-LBL/ DLBCL | No | No | No |
| 56 | M | 117 | DLBCL | No | No | No |
| 59 | M | 119 | DLBCL | No | No | No |
| 65 | M | 106 | No | No | No | No |
| 69 | M | 102 | DLBCL | No | No | Spleen hyperplasia |
| 70 | M | 103 | No | No | No | Thymus hyperplasia |
| 71 | M | 90 | No | No | No | No |
| 1-24-2 | M | 83 | No | Yes | No | No |
| 2-15-2 | F | 140 | Lymphoma | No | No | EMH in liver |
| 2-19-6 | M | 132 | No | No | Pancreas | EMH in Spleen |
| 2-19-2 | F | 143 | No | No | No | EMH in Spleen |
| 3-11-3 | M | 129 | No | No | No | No |
| 3-28-5 | F | 85 | No | Yes | No | EMH in spleen |
| 3-9-7 | M | 129 | No | No | No | No |
| 5-12-3 | F | 99 | Lymphoma | No | No | EMH in Spleen |
| 7-9-9 | F | 116 | No | No | No | No |
| 8-2-6 | M | 120 | No | No | No | No |
| 10-24-7 | F | 130 | No | No | Pancreas | EMH in spleen |
| 10-26-6 | F | 129 | Histiocytic sarcoma | No | No | EMH in spleen/liver |
| 11-10-7 | M | 121 | No | No | No | No |
| 11-7-3 | F | 121 | No | No | No | No |
| 11-29-2 | F | 144 | No | Yes | No | EMH in spleen |
| 12-2-4 | F | 113 | No | No | No | No |
| 12-20-7 | F | 115 | No | No | No | EMH in Spleen |
| 11-9-6 | F | 96 | DLBCL | No | Skin | No |
| 1-19-1 | F | 86 | No | No | Skin | No |
| 11-10-15 | F | 105 | No | No | Skin/Pancreas | No |
| 12-25-6 | M | 111 | No | No | No | Hepatocirrhosis |
| 1-19-5 | F | 109 | Lymphoma | No | No | No |
| 7-22-4 | M | 86 | No | No | No | No |
| 7-22-7 | M | 126 | No | No | Kidney | No |
| 11 | M | 111 | N/A |  |  | Found dead |
| 42 | M | 111 | N/A |  |  | Found dead |
| 43 | M | 107 | N/A |  |  | Found dead |
| 46 | M | 117 | N/A |  |  | Found dead |
| 52 | M | 101 | N/A |  |  | Found dead |

These mice were from published studies (Zhang et al, 2017, Genes & Dev, 31:1243-56 (Ref 16); Yang et al, 2017, PNAS, 114 (43) 11500-11505 (Ref 29)).

T-LBL: Thymic lymphoblastic lymphoma; DLBCL: Diffuse large B-cell lymphoma; N/A: not applicable; EMH: extramedullary hematopoiesis.
