# Supplementary material for: Ferredoxin 2 Is Critical for Tumor Suppression and Lipid Homeostasis but Dispensable for Embryonic Development
Source: Am J Pathol. 2024 Dec 26;195(4):705–16. doi: 10.1016/j.ajpath.2024.12.002 (PMC13169309; doi:10.1016/j.ajpath.2024.12.002)
Supplement: Supplemental Table S2 [file mmc4.docx]

**Supplemental Table S2.** *Fdxr^+/-^* mice - survival time, tumor spectrum and other abnormalities (n=31).

| **ID** | **Sex** | **Survival (Wks)** | **Tumor** | **Other abnormalities** |
| --- | --- | --- | --- | --- |
| 1 | M | 123 | DLBCL in thymus, salivary, lung, liver, pancreas, and spleen | Liver steatosis and hepatitis |
| 6 | M | 119 | Not applicable | Found dead |
| 14 | M | 100 | Hepatocellular carcinoma; Spleen angiosarcoma | Liver steatosis |
| 17 | M | 115 | Lung adenocarcinoma | Liver steatosis; Thymus hyperplasia |
| 18 | M | 98 | Not applicable | Found dead |
| 24 | M | 116 | Lung carcinoma | Liver necrosis and hepatitis |
| 27 | M | 115 | T-LBL in thymus | Liver hepatitis; Lung chronic inflammation |
| 21 | M | 114 | Liver hemangioma; DLBCL in adrenal gland and spleen | EMH in spleen |
| 33 | M | 112 | T-LBL in thymus, kidney and spleen | No |
| 31 | M | 102 | T-LBL in thymus, salivary, kidney, pancreas and intestine | No |
| 36 | M | 125 | Liver angiosarcoma | Mildly enlarged spleen |
| 38 | M | 104 | No | Liver steatosis; large cell dysplasia in liver and spleen |
| 57 | M | 106 | No | Liver steatosis; Kidney inflammation |
| 60 | M | 115 | Liver hemangioma; Lung adenocarcinoma | Liver steatosis and chronic hepatitis |
| 15 | F | 102 | T-LBL in Thymus; Spleen angiosarcoma | Large cell hyperplasia in liver |
| 32 | F | 92 | Fibrosarcoma and lymphoma on left shoulder; lymphoma in salivary gland, spleen and lung | Liver chronic hepatitis |
| 63 | F | 86 | DLBCL in liver, thymus, spleen and pancreas | Liver steatosis and hepatitis; Lymphocytic sialadenitis; Endometrial polyp |
| 73 | F | 68 | DLBCL in spleen and in kidney | Lymphocytic sialadenitis; Spleen hyperplasia |
| 40 | F | 100 | DLBCL in spleen | Liver steatosis |
| 41 | F | 39 | Myeloid sarcoma in spleen; DLBCL in liver, spleen, kidney and intestine; Ovarian adenoma | Liver chronic hepatitis |
| 47 | F | 60 | No | Liver steatosis |
| 48 | F | 100 | High grade sarcoma in liver, ovary, uterus, and abdomen; DLBCL in spleen | Hepatitis; Liver steatosis |
| 35 | F | 77 | T-LBL in thymus | Large cell hyperplasia |
| 51 | F | 113 | Thymus adenoma; DLBCL in salivary gland | Liver steatosis and hepatitis |
| 53 | F | 90 | DLBCL in liver, thymus, lung, pancreas and ovary | Liver steatosis |
| 54 | F | 102 | Lung adenocarcinoma | Liver steatosis |
| 58 | F | 97 | Hepatocellular carcinoma; Spleen myeloid sarcoma, lymphoma and hemangioma | Liver steatosis; Intestine appendiceal epiploica |
| 29 | F | 108 | Salivary gland and kidney plasmacytoma | Thymus hyperplasia |
| 61 | F | 100 | DLBCL in kidney | Thymus, spleen hyperplasia |
| 4 | F | 114 | DLBCL in thymus, kidney, ovary, pancreas, shoulder, and abdominal lymph nodes. | Liver steatosis with inflammation; pancreas islet cell hyperplasia |
| 19 | F | 116 | DLBCL in liver, pancreas, spleen, kidney, and ovary | Hepatitis and Pneumonia |

T-LBL: T-cell lymphoblastic lymphoma; DLBCL: Diffuse large B-cell lymphoma; EMH: extramedullary hematopoiesis.

*Fdxr*^+/-^ mice were from published studies (Zhang et al, 2017, Genes & Dev, 31:1243-56 (Ref 16); Zhang et al, 2019, Oncogene, 38:6256-69 (Ref 30)).
