# Supplementary material for: Ferredoxin 2 Is Critical for Tumor Suppression and Lipid Homeostasis but Dispensable for Embryonic Development
Source: Am J Pathol. 2024 Dec 26;195(4):705–16. doi: 10.1016/j.ajpath.2024.12.002 (PMC13169309; doi:10.1016/j.ajpath.2024.12.002)
Supplement: Supplemental Table S3 [file mmc5.docx]

| **Supplemental Table S3.** Fdx2+/- mice (n=33) - survival time, tumor spectrum, steatosis, inflammation, and other abnormalities. | | | | |  |
| --- | --- | --- | --- | --- | --- |
| ID | Sex | Survival (Wks) | Tumor | Inflammation | Steatosis  /Steatohepatitis |
| 79 | F | 52 | Pleomorphic sarcoma | Kidney/Liver/Salivary gland/Pancreas | No |
| 10-27-7 | M | 113 | DLBCL in spleen and lymph nodes | Salivary gland/Liver | No |
| 5-16-18 | F | 102 | DLBCL in liver, spleen, lung, and kidney; focal hepatocellular carcinoma | Liver/Salivary gland/GI tract/Brown fat | Yes |
| 25 | M | 44 | - | Salivary gland | No |
| 35 | M | 68 | - | Salivary gland/ Lung/ Liver/Pancreas/Kidney | Yes |
| 10-27-3 | F | 82 | - | Salivary gland/Liver | Yes |
| 11-1-2 | M | 49 | - | Salivary gland | Yes |
| 24 | M | 86 | Tubular adenoma of colon | Salivary gland | Yes |
| 40 | F | 121 | DLBCL in thymus, lung, kidney and stomach | Liver//Lung/Salivary gland | No |
| 5-20-4 | F | 125 | Large B cell lymphoma in spleen, lungs and lymph nodes | Kidney/Liver/Salivary gland | No |
| 59 | F | 108 | Infarct hematoma | Lung | No |
| 16 | M | 103 | - | Salivary gland/Lung/Liver/Pancreas/ Kidney/Brown fat | Yes |
| 10-27-16 | F | 111 | DLBCL in spleen and pancreas; Hepatocellular carcinoma | Salivary gland/Lung/Liver/ Kidney/Abdominal Fat | Yes |
| 6-13-4 | M | 72 | - | Salivary gland/Abdominal Fat | Yes |
| 5-20-7 | F | 75 | - | Salivary gland/Kidney/ Abdominal Fat | No |
| 5-7-16 | F | 86 | - | Liver/Salivary gland/ Abdominal and Brown Fat | No |
| 4-14-39 | F | 106 | - | Liver/Salivary gland/ Abdominal and Brown Fat | Yes |
| 35 | F | 68 | - | Liver/Salivary gland/ Lung/Kidney/ Abdominal and Brown Fat | Yes |
| 10-27-4 | F | 82 | - | Salivary gland/Abdominal fat | Yes |
| 11-23-26 | F | 93 | Lung adenocarcinoma | Kidney/Salivary gland/Liver/ Abdominal Fat | Yes |
| 25 | M | 43 | Focal branch alveolar carcinoma | Salivary gland/ Brown fat | No |
| 11-1-19 | M | 81 | Lymphoma | Liver/Kidney/Salivary gland/ Abdominal Fat | No |
| 69 | F | 124 | - | Salivary gland/Liver/ Kidney/ Abdominal and Brown Fat | Yes |
| 73 | F | 80 | - | Salivary gland/Lung/ Kidney/ Abdominal Fat | No |
| 10-27-1 | M | 107 | DLBCL and Lymphoma in spleen and GI tract | Liver/Kidney/Salivary gland/ Lung/Abdominal Fat | Yes |
| 74 | F | 70 | DLBCL in liver and spleen | Kidney/Salivary gland/ Abdominal Fat | No |
| 5-20-1 | M | 73 | - | Kidney/Salivary gland/ Lung/ Abdominal Fat | No |
| 56 | F | 73 | - | Kidney/Salivary gland/ Lung/ Liver/Pancreas/Abdominal Fat | Yes |
| 33 | F | 118 | DLBCL in spleen, lung, liver and lymph nodess | - | No |
| 10-27-5 | M | 102 | - | Liver/Kidney/Salivary gland/ Lung/Pancreas/Abdominal Fat | No |
| 11-23-21 | F | 116 | Granulosa cells tumor (Sex cord stroma tumor) | Kidney/Salivary gland/ Pancreas/Brown Fat | No |
| 5-20-6 | F | 75 | - | Kidney/Salivary gland/Abdominal Fat | No |
| 11-1-18 | M | 88 | - | Kidney/Salivary gland/Liver/ Abdominal Fat | Yes |

DLBCL: Diffuse large B-cell lymphoma; N/A: not applicable; EMH: extramedullary hematopoiesis.
