# Supplementary material for: Ferredoxin 2 Is Critical for Tumor Suppression and Lipid Homeostasis but Dispensable for Embryonic Development
Source: Am J Pathol. 2024 Dec 26;195(4):705–16. doi: 10.1016/j.ajpath.2024.12.002 (PMC13169309; doi:10.1016/j.ajpath.2024.12.002)
Supplement: Supplemental Table S4 [file mmc6.docx]

**Supplemental Table S4.** Fdx2-/- mice (n=19) - survival time, tumor spectrum, steatosis, inflammation, and other abnormalities.

| ID | Sex | Survival (Wks) | Tumor | Inflammation | Steatosis/  Steatohepatitis |
| --- | --- | --- | --- | --- | --- |
| 32 | F | 93 | - | Kidney/Salivary gland/Pancreas | No |
| 11-1-15 | M | 88 | - | Salivary gland/Kidney | No |
| 2 | F | 76 | - | Salivary gland/Liver | No |
| 65 | F | 102 | Adenocarcinoma, possibly poorly differentiated breast invasive ductal carcinoma | Kidney | No |
| 6-13-2 | F | 118 | Large B cell lymphoma in thymus and lymph node | Salivary gland | No |
| 3-22-1 | F | 81 | - | - | No |
| 3-22-5 | F | 104 | Lipoma with necrosis and chronic inflammation | Salivary gland/Lung/Liver/Pancreas/Kidney  /Abdominal and Brown Fat | Yes |
| 5-20-2 | F | 76 | - | Salivary gland/Kidney/Abdominal Fat | No |
| 6-13-1 | F | 95 | - | Liver/Pancreas/Salivary gland/Kidney/Abdominal and brown fat | No |
| 4-14-43 | F | 52 | Histiocytic sarcoma in liver, kidney, spleen and lymph node | Lung | No |
| 33 | F | 97 | DLBCL in lymph nodes | Salivary gland/Liver/Pancreas | Yes |
| 11-23-20 | F | 101 | - | Salivary gland/Liver/Pancreas/ Kidney/ Abdominal and Brown fat/Skin | No |
| 5-20-10 | M | 84 | DLBCL in lymph node on GI | Salivary gland/Pancreas/ Abdominal Fat | No |
| 6-13-5 | F | 87 | Papillary adenocarcinoma | Liver/Salivary gland/Pancreas/Brown Fat | Yes |
| 6-13-10 | M | 88 | DLBCL in lymph nodes on GI | Salivary gland/Lung/Liver/Pancreas/ Brown fat/Brain | No |
| 6-13-30 | M | 81 | - | Salivary gland/Lung/Liver/Pancreas/Brown Fat | Yes |
| 4-14-40 | M | 75 | - | Salivary gland/Liver/Abdominal Fat | Yes |
| 5-20-8 | M | 75 | DLBCL in lymph node on GI | Salivary gland/Pancreas/Abdominal Fat | No |
| 5-20-12 | M | 84 | - | Salivary gland/Kidney/Brown Fat | No |

DLBCL: Diffuse large B-cell lymphoma; GI: Gastrointestinal tract.
